# Comparison of Sulphate-reducing Bacterial Communities in Japanese Fish Farm Sediments with Different Levels of Organic Enrichment

**DOI:** 10.1264/jsme2.ME11278

**Published:** 2012-02-22

**Authors:** Ryuji Kondo, Yumi Mori, Tomoko Sakami

**Affiliations:** 1 Department of Marine Bioscience, Fukui Prefectural University, Gakuen-cho, Obama, Fukui 917–0003, Japan; 2 Tohoku National Fisheries Research Institute, Fisheries Research Agency, Shinhama-cho, Shiogama, Miyagi 985–0001, Japan

**Keywords:** fish farm sediments, dissimilatory sulphite reductase gene, organic gradient

## Abstract

Fish farm sediments receive a large amount of organic matter from uneaten food and fecal material. This nutrient enrichment, or organic pollution, causes the accumulation of sulphide in the sediment from the action of sulphate-reducing bacteria (SRB). We investigated the effect of organic enrichment around coastal fish farms comparing the SRB community structure in these sediments. Sediment samples with different levels of organic pollution classified based upon the contents of acid-volatile sulphide and chemical oxygen demand were collected at three stations on the coast of western Japan. The SRB community composition was assessed using PCR amplification, cloning, sequencing and phylogenetic analysis of the dissimilatory sulphite reductase β-subunit gene (*dsrB*) fragments using directly extracted sediment DNA. Sequencing of the cloned PCR products of *dsrB* showed the existence of different SRB groups in the sediments. The majority of *dsrB* sequences were associated with the families *Desulfobacteraceae* and *Desulfobulbaceae*. Clones related to the phylum *Firmicutes* were also detected from all sediment samples. Statistical comparison of sequences revealed that community compositions of SRB from polluted sediments significantly differed from those of moderately polluted sediments and unpolluted sediments (LIBSHUFF, *p*<0.05), showing a different distribution of SRB in the fish farm sediments. There is evidence showing that the organic enrichment of sediments influences the composition of SRB communities in sediments at marine fish farms.

Aquaculture is a major industry worldwide and has led to concern about the impact of fish farming on previously pristine marine environments. Fish farm sediments receive a large amount of organic waste due to uneaten food and fecal materials. This organic enrichment of sediment influences the biogeochemical processes of benthic microbial communities (1, 13, 19); and the anaerobic conditions result in the accumulation of hydrogen sulphide. Dissimilatory reduction of sulphate by sulphate-reducing prokaryotes is responsible for the production and accumulation of hydrogen sulphide in marine sediments. The process of sulphate reduction is anaerobic terminal mineralization of organic matter in coastal marine sediments, degrading up to 50% of all organic matter in coastal marine sediments (17).

The use of molecular techniques to describe microbial populations in natural communities has attracted considerable interest. Direct amplification of the functional genes as well as the 16S rRNA gene (16S rDNA) from environmental samples using PCR has proven to be an attractive technique for characterising members of complex microbial assemblages. Clone library analysis has been performed to investigate the changes in microbial communities with reference to marine fish farming (3, 19, 35). 16S rDNA library clones show that fish farm sediments are dominated by α-,γ-, δ-, and ɛ-*Proteobacteria*, *Acidobacteria*/*Holophaga*, *Bacteroidetes* and *Planctomycetes* where the phylogenetic compositions of the 16S rDNA clone libraries differed among the sites or levels of organic enrichment. In these clone library analyses, δ-*Proteobacteria* were found and selected from within highly enriched fish farm sediments. Most of the clone sequences in the nutrient-rich fish farm sediments were phylogenetically related to sulphate-reducing bacteria (SRB) (3, 19), suggesting that SRB abundance increases with increasing organic enrichment. Using quantitative PCR, we found differences in SRB densities in fish farm sediments with different enrichment levels (18). Further, molecular analyses of the gene coding for the α-subunit (*dsrA*) and/or β-subunit (*dsrB*) of dissimilatory sulphite reductase retrieved from sediment samples showed the differential distribution of SRB groups in sediments along the coasts of Japan and South Korea (Kondo *et al.*, unpublished data); and in a UK estuary where increased organic matter gradients occurred (22, 25). Thus, SRB diversity and/or specific SRB densities may be used as a biological indicator to assess pollution levels in sediments of marine fish farms as an adjunct to chemical analysis of pollutants.

Here we investigated the differences in community compositions of SRB in sediments beneath aquaculture fish cages having different pollution levels. Culture-independent clone library analysis of *dsrB* was employed in combination with chemical analyses. We showed relationships between the SRB communities and organic enrichment in the sediments.

## Materials and Methods

### Sediment sampling

From July to September 2008, sediment samples were collected at three sites: Gokasho Bay (Gokasho A2) in Mie Prefecture, Shitaba Bay (Shitaba S-7) in Ehime Prefecture, and the Yatsushiro Sea (Yatsushiro A) in Kagoshima Prefecture (Table 1). Surface sediments were collected using an Ekman-Birge bottom sampler (Rigo Co. Ltd., Saitama, Japan) equipped with a small core. From these core samples, subsamples of surface sediment were taken from the 0–4 cm depth horizon. Replicate or more cores were taken from each site and the sediment samples were gathered in sterile sampling bags (Fisher Scientific, Pittsburgh, PA, USA). Samples for sulphide analysis were fixed using zinc acetate powder while at sea. The samples were stored on ice for transport to the laboratory.

### Chemical analyses

Acid-volatile sulphides (AVSs) in the sediments were separated using steam distillation under acid conditions and trapped in a 10% (w/v) zinc acetate solution. The trapped sulphides were determined using the methylene-blue method and the data were recorded using spectrophotometry (21). Chemical oxygen demand (COD) was measured using the standard Mn-COD method (6). Moisture content was determined, drying the sediment samples at 60°C until a constant weight was obtained.

### Nucleic acid extraction

Sediment samples were mixed well in sterile plastic bags immediately prior to weighing out 0.5 g sediment sample into a Lysing Matrix E with a small spatula baked at 260°C for 2 h. Total nucleic acids were extracted from the 0.5 g sediment sample using a FastDNA Spin Kit for Soil (MP Biomedicals LLC, Solon, OH, USA) with some modification (26). The extracted DNA was diluted 10-fold with TE buffer (10 mM Tris-HCl, 1 mM EDTA, pH 8.0) to reduce potential inhibitors of PCR from sediment samples and to adjust the amount of DNA for PCR amplification (46). One microlitre of the dilution was used for PCR.

### Quantitative PCR to enumerate SRB

Quantitative real-time PCR (qPCR) was performed as described by Kondo *et al.*(26). Briefly, the primers DSR1F+ (5′-ACSCACTGGAAGCACGGCGG-3′) and DSR-R (5′-GTGGMRCCGTGCAKRTTGG-3′) were used (22, 26). The reaction mixture (50 μL) was: 25 μL Master Mix (2× QuantiTect SYBR Green; Qiagen, Hilden, Germany), 2 μL 25 mM MgCl_2_, 1 μL each primer 20 μM and 1 μL template DNA extract. qPCR was performed using an iCycler iQ system (Bio-Rad Laboratories, Hercules, CA, USA): 15 min at 95°C for initial denaturation; and 40 cycles: 15 s at 94°C, 30 s at 60°C and 30 s at 72°C.

### PCR amplification, cloning and sequencing of dsrB

For assessing the SRB community compositions, we chose a portion of the *dsrB* gene to generate longer PCR products than the *dsrA* gene used for qPCR. Amplification of *dsrB* gene fragments was performed using the primer set DSRp2060F (5′-CAACATCGYCAYCCCAGGG-3′; 10) and DSR4R (5′-GTGTAGCAGTTACCGCA-3′; 43) as described by Foti *et al.*(8). The yield and quality of the PCR products were examined on 1% (w/v) agarose gel stained with ethidium bromide.

After PCR amplification, unpurified *dsrB* PCR products were cloned using a TA Cloning Kit (Invitrogen, Carlsbad, CA, USA) with the pCR II vector into *Escherichia coli* INVαF′ competent cells according to the manufacturer’s instructions. Clones containing an insert were randomly selected from each library for sequencing. The clone inserts were re-amplified using the vector primers, M13 forward and reverse (25 cycles: 94°C for 30 s, 50°C for 30 s, and 72°C for 30 s); and the PCR products were purified using a Wizard SV Gel and PCR Clean-Up System (Promega, Madison, WI, USA) according to the manufacturer’s instructions. Selected clones were sequenced using a DNA sequencer model ABI Prism 3130 Genetic Analyzer (Applied Biosystems, Foster City, CA, USA) employing M13 forward and/or reverse primers.

### Phylogenetic analysis

Primer sequences were excluded from phylogenetic analyses. We compared our sequencing data to DNA databases using the BLAST programme. The *dsrB* sequences were translated using the BioEdit software package, version 7 (12). Using Clustal X (41), the translated amino acid sequences were aligned compared to representative prokaryote DsrB sequences from the DDBJ/EMBL/GenBank databases. Neighbor-joining analysis was performed using the MEGA software package, version 4 (40). The confidence limits for the tree topology were estimated using bootstrap analysis (7) with 1,000 replications.

### Statistical analyses and sequence population diversity

Sequences were grouped into operational taxonomic units (OTUs) using the software DOTUR (37). Here, clones with sequence similarity greater than 85% were considered to represent the same OTU (25), and for the purposes of calculating diversity statistics, Simpson’s and Shannon-Wiener indexes were calculated using DOTUR.

Coverage (*C*) was calculated using the following formula *C*=1− (*n*_1_/*N*), where *n*_1_ is the number of OTUs that occurred only once in the clone library and *N* is the total number of clones analysed (31). The phylogenetic compositions of the libraries were compared using the Sorensen similarity index, *Cs*=2*j*/(*a*+*b*) where *j* is the number of OTUs common to both samples and *a* and *b* are the numbers of OTUs in libraries A and B, respectively (30). Differences in statistical significance in the compositions of library pairs were tested using the LIBSHUFF programme (38).

### Nucleotide sequence accession numbers

The nucleotide sequences reported in this study are deposited in the DDBJ under accession numbers AB631054–AB632344.

## Results and Discussion

### Sediment characteristics

We obtained fish farm sediments having different organic enrichment levels or organic contamination and AVS content. The COD and AVS contents in the sediment at each sampling site are shown in Table 1. COD and AVS in the sediment at all sites were significantly different (*t*-test, *p*<0.05). The data indicate that organic enrichment and anoxic conditions may develop in highly organic and AVS-rich Gokasho A2 sediment. The pollution levels of fish farm sediments were classified by measuring AVS and COD according to the environmental standard for aquaculture of the Japan Fisheries Resource Conservation Association (15, 16): unpolluted (AVS less than 0.2 mg [g dry sediment]^−1^ and COD less than 20 mgO_2_ [g dry sediment]^−1^), moderately polluted (AVS ranging from 0.2 to 1.0 mgS [g dry sediment]^−1^ and COD ranging from 20 to 30 mgO_2_ [g dry sediment]^−1^) and polluted (AVS more than 1.0 mgS [g dry sediment]^−1^ and COD more than 30 mgO_2_ [g dry sediment]^−1^). According to the environmental standard for aquaculture, Gokasho A2 sediments were classified as polluted, Shitaba S-7 were classified as moderately polluted, and Yatsushiro A sediments were unpolluted.

We determined the density of SRB using qPCR to compare the sediment samples and used the same samples for chemical analyses. The *dsrA* of SRB was detected using qPCR with DSR1F+ and DSR-R primers. High densities of SRB ranging from 9.6±1.1×10^8^ to 2.2±0.2×10^9^ cells (g dry sediment)^−1^ were detected in the sediments (Table 1), which is within the range of values reported for other marine and estuarine sediments (18, 22, 28). Statistical analysis indicated the SRB densities were not significantly different, with the exception of the Shitaba S-7 and Yatsushiro A samples (*t*-test, *p*<0.05). Previously, we demonstrated that SRB densities did not exceed 10^9^ cells (g dry sediment)^−1^ in unpolluted sediments (18). SRB densities in the unpolluted sediments at Yatsushiro A averaged 9.6±1.1×10^8^ cells (g dry sediment)^−1^, not exceeding 10^9^ (g dry sediment)^−1^, while no clear distribution pattern of SRB was observed in the sediments at the three sites investigated.

### Phylogenetic analysis

PCR amplification of *dsrB* using the primer set DRSp2060F and DSR4R resulted in products of the predicted size of *ca.* 350 bp from all fish farm sediment samples. We sequenced 1,291 clones (489 clones from Gokasho A2, 471 clones from Shitaba S-7 and 334 clones from Yatsushiro A). The 1,291 clones had 932 unique sequences clustered into 217 OTUs using our definition of >85% sequence identity. One hundred eighteen OTUs were detected in the Gokasho A2 sediments; 89 OTUs in Shitaba S-7 and 101 OTUs in Yatsushiro A (Table 2).

**Fig. 1:**
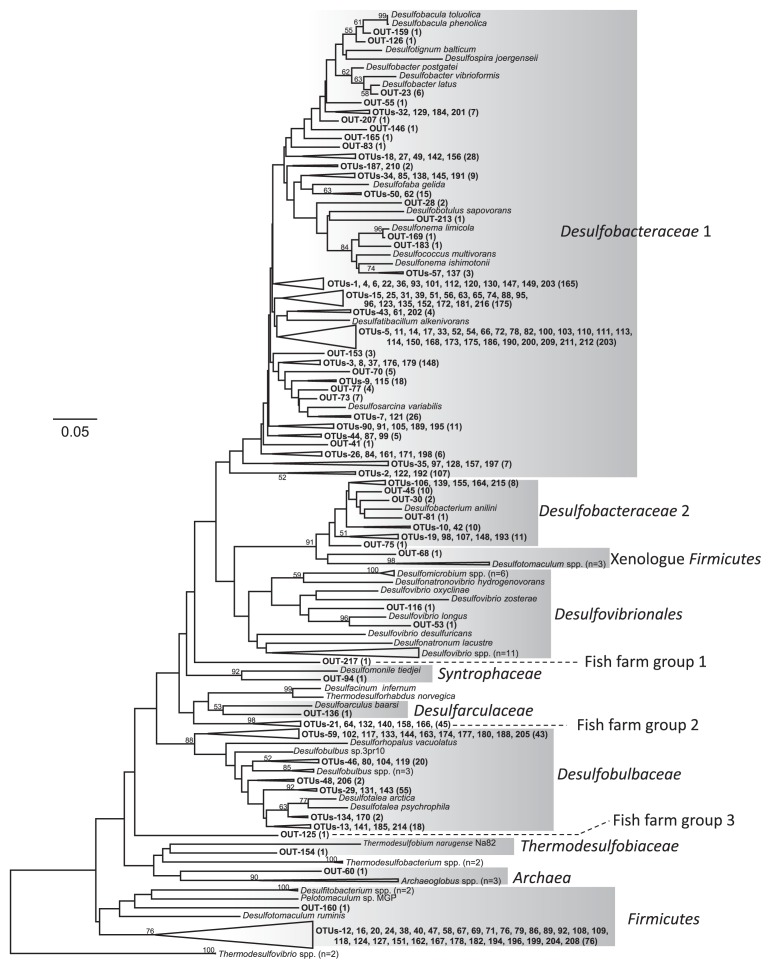
Phylogenetic tree from the translated amino acid sequences of PCR-amplified *dsrB* genes retrieved from fish farm sediments along the coast of Japan. Environmental sequences determined in this study are shown in bold. Bootstrap values based on 1,000 replicates are shown for branches with more than 50% support. Scale bar corresponds to 5% estimated sequence divergence. Numbers in parentheses are the numbers of clones.

To obtain a reliable description of the phylogenetic relationships of the SRB populations of fish farm sediments, we included the most characterized sequences of SRB isolated cultures available from the database. Although differences in tree topologies were obtained between the partial sequences from almost the entire DsrAB sequences (9, 20), similar ordering of taxa was found between these analyses. Neighbor-joining analysis revealed 13 lineages in the cloned *dsrB* sequences. Some were related to cultured SRB, but most almost certainly represent undescribed SRB (Fig. 1).

More than 75% of the clones recovered from all three sediment libraries were related to members of *Desulfobacteraceae* group 1, *e.g.*, genera *Desulfobacter*, *Desulfococcus*, *Desulfosarcina* and *Desulfonema*. Additionally, clones related to *Desulfobacterium anilini* within the *Desulfobacteraceae* family (referred to as *Desulfobacteraceae* group 2) were also detected from all sites at somewhat lower frequencies. These organisms completely oxidise a diverse range of organic compounds, including acetate and other short chain fatty acids, to CO_2_. Most of these sequences were closely related to the DSR gene from uncultured SRB found in the environmental samples. These sequences have been abundantly recovered from surface sediments in many locations: in the surface sediments of Aarhus Bay, Denmark (42), a New England salt marsh (2), the German Wadden Sea (32), the Colne estuary, UK (22, 25) and the Japanese meromictic Lake Suigetsu (23, 24). *Desulfobacter* is reported to be the predominant active SRB genus in marine sediments (36). Thus, SRB related to complete oxidisers from the *Desulfobacteraceae* family are abundantly present and must play an important role in the anaerobic mineralisation of organic matter and in the production of sulphide along the coastal sea.

The second most abundant sequences were related to incomplete oxidisers from the family *Desulfobulbaceae* from the genera *Desulfotarela*, *Desulforhopalus* and *Desulfobulbus*. Cloned sequences from this group were closely related to environmental sequences retrieved from marine environments as well as limnic environments (2, 24, 27, 29, 33, 34, 47). The *Desulfobalbaceae* are physiologically diverse capable of using alternatives to sulphate as electron acceptors, the disproportionation of sulphur and sulphur oxyanions, and sulphate-free growth via fermentation; therefore, this group of SRB appears to play a significant role in the production of sulphide in coastal marine sediments and supplies electron donors for the complete oxidising SRB.

A group of 77 *dsrB* clones were phylogenetically related to members of the genera of the *Desulfotomaculum* and *Pelotomaculum*, the *Firmcutes* group. This Gram-positive group of organisms grows optimally in low salt concentrations and thus seems to inhabit primarily freshwater environments or other aqueous environments with relatively low salt concentrations (45). Using phylogenetic analyses of *dsrAB* genes, we found that sequences related to *Desulfotomaculum* were abundantly recovered from low saline environments (4, 5, 24, 27). Of the clones affiliated in the *Firmicutes* group, 57% of the clones were recovered from the polluted, nutrient-rich, shallow Gokasho A2 location in the innermost part of Gokasho Bay.

### Comparison of SRB diversity in the clone library

**Fig. 2:**
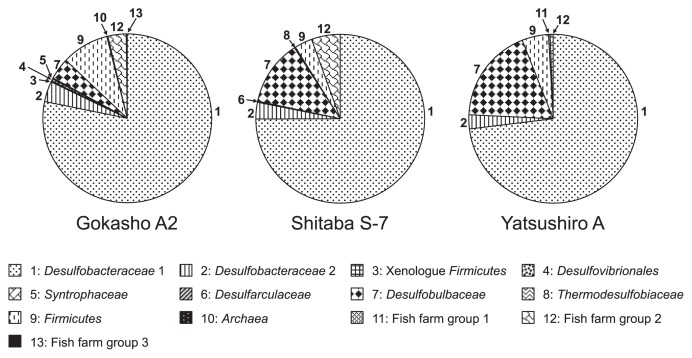
Spatial distribution of *dsrB* clones in libraries from sediment samples collected at three fish farms

The relative abundance of the 13 different groups in the libraries was calculated for all three samples (Fig. 2 and Table 3). Five of these groups were detected at all three sites. Two of these groups, *Desulfobacteraceae* group 1 and group 2, were evenly distributed throughout the fish farm sediments. Clones from the *Desulfobacteraceae* group 1 and group 2 represented a greater proportion of the libraries from the polluted Gokasho A2 site, as did the clones from the *Firmicutes* group; while clones related to the *Desulfobulbaceae* family were more abundant in the unpolluted sediment of Yatsushiro A than at the other two sites. Clones from fish farm group 2 were recovered at relatively higher frequencies from the polluted Gokasho A2 and moderately polluted Shitaba S-7 than at the unpolluted Yatsushiro A site. No more than two clones were detected at any of the sites from each of the remaining eight groups: the xenologue *Firmicutes* group, the *Desulfovibrionales* group, the *Syntrophaceae* group, the *Desulfoarculaceae* group, the *Thermodesulfobiaceae* group, the *Archaea* group and the fish farm group 1 and group 3.

**Fig. 3:**
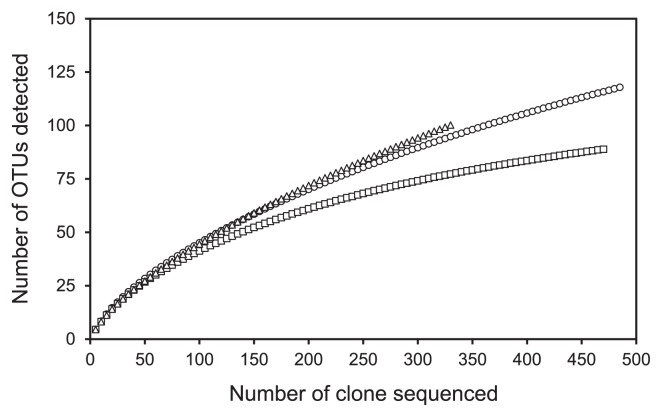
Rarefaction curves generated for *dsrB* in clone libraries from samples collected at Gokasho A2 (circle), Shitaba S-7 (square) and Yatsushiro A (triangle).

Using the 85% similarity cut-off, coverage values for each library were from 80.5 to 93.0%, and rarefaction analysis (Fig. 3) showed that the libraries were reasonably well sampled for diversity (31). The Simpson’s indexes were high, ranging from 0.946 at Yatsushiro A to 0.955 at Gokasho A2, suggesting that all sediments were considerably diverse. This index, the 1-λ, in Table 2, may be thought of as an “evenness index” where 1.0 approaches the maximum values. The Simpson’s and Shannon indices were slightly higher for the polluted Gokasho A2 site than for the other two sites (Table 2). This indicates that polluted sediments from fish farms were more diverse than the moderately polluted or unpolluted sediments of the other fish farms. Differences in SRB diversity were observed between bottom sediments and water columns in the meromictic Lake Suigetsu (24), and contrasted with two mudflats in the Seine estuary in France (27), and among the surface sediments along environmental gradients in the River Colne estuary in UK (25). SRB diversity was found to differ slightly among the fish farm sediments, possibly depending on the depth of the water, distance from land or different pollution levels, and/or a combination of the three. Apart from these factors, temperature is a possible factor influencing the diversity or community composition of SRB in sediments. We did not measure the sediment temperature, but the difference in the water temperature 1 m above the bottom between Gokasho A-2 and Shitaba S-7 was 2.2°C. Thus, it did not appear to alter the community compositions of SRB in the fish farm sediments.

The Sorensen similarity indexes from the SRB OTU populations in the sediment libraries were from 0.438 to 0.537 (Table 4). The significance of these differences between the clone libraries was examined using LIBSHUFF statistics. LIBSHUFF analysis showed that the library from the polluted Gokasho A2 site was significantly different from those from the moderately polluted Shitaba S-7 and the unpolluted Yatsushiro A. These pairwise comparisons show that the Shitaba S-7 library was not different from the Yatsushiro A library (Table 4). High COD and AVS contents were detected in the sediment from Gokasho A2. Although most of the major clones obtained in this study were commonly distributed among the three fish farm sediment libraries, some clones were detected from only one of these libraries (Table S1). For example, 35 clones occurring in OTU-5 were retrieved only from the polluted Gokasho A2 library. Clones in OTU-7 were recovered primarily from the Gokasho A2 library; however, a single clone was isolated from the Shitaba S-7 library. Cloned sequences in these OTUs were related to *dsrB* clone sequences recovered from various anoxic environments, including organic-rich, sulphidogenic marine sediments (2, 11, 16, 28, 39, 44). These environmental clones are reported to belong to the *Desulfobacteraceae* family. While no clone sequences occurred only in the moderately polluted Shitaba S-7 sediment or in the unpolluted Yatsushiro A sediment, clones in OTUs-37 and -103 were commonly retrieved from the Shitaba S-7 and Yatsushiro A libraries, respectively (Table S1). Differences in the SRB community compositions among fish farm sediments may be caused by different pollution levels, as indicated using AVS and COD values. Some *dsrB* clones were selected for within polluted or unpolluted fish farm sediments. In our previous study on the distribution of SRB in puffer fish farm sediments, we used quantitative PCR to detect differences in SRB densities in fish farm sediments with different levels of organic pollution. This suggested that SRB cell densities may be used as a biological indicator to assess pollution levels in sediments of marine fish farms (18). Moreover, our data shown here suggest that these SRB may be used as biological indicators to assess pollution levels in sediments of marine fish farms as an adjunct to chemical analyses, as shown in our COD and AVS data.

## Conclusions

We assessed the SRB community composition in fish farm sediments with different levels of organic enrichment pollution. Using a *dsrB* clone library constructed from fish farm sediments, most clones fell into four lineages of sulphate-reducing prokaryotes: *Desulfobacteraceae*, *Desulfobulbaceae*, *Firmicutes*, and a deeply branched group in the DsrB phylogenetic tree with no representatives from previously isolated SRB. The compositions of the *dsrB* clone libraries were significantly different between the polluted and moderately polluted/unpolluted sediments. The *dsrB* sequences related to the *Desulfobulbaceae* family were recovered with relatively higher frequency from unpolluted sediments than polluted sediments. In contrast, the most abundant clone sequences from polluted sediment samples were grouped within the *Desulfobacteraceae* family and the *Firmicutes* phylum. Our data suggest that organic enriched or polluted sediments can influence the composition of SRB communities. A general SRB indicator of organic pollution was not identified; however, several *dsrB* gene sequences were highly prevalent in the polluted sediments of fish farms. Therefore, the data presented here show a complex distribution of SRB among the fish farms and these data show that SRB may be used as a statistically significant biological indicator to assess pollution levels in marine fish farm sediments.

## Supplementary material



## Figures and Tables

**Table 1:** Sampling locations and sediment characteristics of fish farm sediment samples

Sampling site	Longitude (E)	Latitude (N)	Date of sampling	Depth (m)	Water temperature^a^ (°C)	DO^a^ (mg L^−1^)	COD (mg O_2_ [g dry sediment]^−1^)	AVS (mg S [g dry sediment]^−1^)	SRB (cells [g dry sediment]^−1^)
Gokasho A2	136°39′	34°19′	17 Jul 2008	17	19.4	4.23	65.4±1.5	1.54±0.21	1.8±0.3×10^9^
Shitaba S-7	132°26′	33°10′	9 Sep 2008	52	21.6	4.21	26.0±1.6	0.27±0.02	2.2±0.2×10^9^
Yatsushiro A	130°14′	32°14′	9 Sep 2008	41	ND^b^	ND^b^	15.1±0.2	0.02±0.00	0.9±0.1×10^9^

a Measured at a water depth 1 m above the bottom.

b ND, not determined.

**Table 2:** Diversity of *dsrB* fragments from fish farm sediments estimated using the Shannon and Simpson diversity indexes computed with the DOTUR programme

Sampling site	Number of clone sequenced	Number of OTU detected	Coverage (%)	Shannon Index^a^ (*H*′)	Simpson^b^ 1-λ
Gokasho A2	486	118	86.6	3.82 (3.69, 3.95)	0.955
Shitaba S-7	471	89	93.0	3.62 (3.50, 3.74)	0.948
Yatsushiro A	334	101	80.5	3.66 (3.50, 3.82)	0.946

a Shannon Index. A high number is more diverse. Numbers in parentheses are lower and upper 95% confidence intervals for the Shannon Index.

b Simpson Index. A high number is more diverse.

**Table 3:** Assignment of *dsrB* clones from sediment samples from fish farms

Phylogenetic affiliations^a^	Fish farm *dsrB* OTU	Number of clones in a library		
		Gokasho A2	Shitaba S-7	Yatsushiro A
*Desulfobacteraceae* 1	1–9, 11, 14, 15, 17, 18, 22, 23, 25–28, 31–37, 39, 41, 43, 44, 49–52, 54, 55–57, 61–63, 65, 66, 70, 72–74, 77, 78, 82–85, 87, 88, 90, 91, 93, 95–97, 99–101, 103, 105, 110–115, 120–123, 126, 128, 129, 130, 137, 138, 142, 145–147, 149, 150, 152, 153, 156, 157, 159, 161, 165, 168, 169, 171–173, 175, 176, 181, 183, 184, 186, 187, 189–192, 195, 197, 198, 200–203, 207, 209–213, 216	380	353	244
*Desulfobacteraceae* 2	10, 19, 30, 42, 45, 75, 81, 98, 106, 107, 139, 148, 155, 164, 193, 215	19	15	9
Xenologue *Firmcutes*	68	1	0	0
*Desulfovibrionales*	53, 116	2	0	0
*Syntrophaceae*	94	1	0	0
*Desulfarculaceae*	136	0	1	0
*Desulfobulbaceae*	13, 29, 46, 48, 59, 80, 102, 104, 117, 119, 131, 133, 134, 141, 143, 144, 163, 170, 174, 177, 180, 185, 188, 205, 206, 214	20	59	61
*Thermodesulfobiaceae*	154	0	1	0
*Firmicutes*	12, 16, 20, 24, 38, 40, 47, 58, 67, 69, 71, 76, 79, 86, 89, 92, 108, 109, 118, 124, 127, 151, 160, 162, 167, 178, 182, 194, 196, 199, 204, 208	44	16	17
*Archaea*	60	1	0	0
Fish farm group 1	217	0	0	1
Fish farm group 2	21, 64, 132, 140, 158, 166	17	26	2
Fish farm group 3	125	1	0	0

a Affiliation of *dsrB* clones as inferred from Fig. 1.

**Table 4:** Comparison of the composition of *dsrB* gene clone libraries from fish farm sediments

	Sorensen similarity index for the libraries from samples collected from fish farm sediments from the following sites	

	Shitaba S-7	Yatsushiro A
Gokasho A2	0.531 (0.001, 0.001)	0.438 (0.001, 0.001)
Shitaba S-7		0.537 (0.111, 0.001)

Values in parentheses are the probabilities that the compositions of the libraries are different as calculated using the LIBSHUFF program (X compared to Y, Y compared to X, where X is the library indicated in the stub and Y is the library in the column head).

## References

[1] Asami H, Aida M, Watannabe K (2005). Accelerated sulfur cycle in coastal marine sediment beneath areas of intensive shellfish aquaculture. Appl Environ Microbiol.

[2] Bahr M, Crump BC, Klepac-Ceraj V, Teske A, Sogin L, Hobbie JE (2005). Molecular characterization of sulfate-reducing bacteria in a New England salt marsh. Environ Microbiol.

[3] Bissett A, Bowman J, Burke C (2006). Bacterial diversity in organically-enriched fish farm sediments. FEMS Microbiol Ecol.

[4] Castro H, Reddy KR, Ogram A (2002). Composition and function of sulfate-reducing prokaryotes in eutrophic and pristine areas of the Florida Everglades. Appl Environ Microbiol.

[5] Chang Y-J, Peacock AD, Long PE, Stephen JR, Mackinley JP, Macnaughton SJ, Hussain AKMA, Saxton AM, White DC (2001). Diversity and characterization of sulfate-reducing bacteria in groundwater at a uranium mill tailings site. Appl Environ Microbiol.

[6] Eguchi M, Ishida Y, Sugita H (2000). Chemical oxygen demand. Microbiological Methods for Assessment of Marine Environments.

[7] Felsenstein J (1985). Confidence limits on phylogenies: an approach using the bootstrap. Evolution.

[8] Foti M, Sorokin DY, Lomans B, Mussman M, Zacharova EE, Pimenov NV, Kuenen JG, Muyzer G (2007). Diversity, activity, and abundance of sulfate-reducing bacteria in saline and hypersaline soda lakes. Appl Environ Microbiol.

[9] Friedrich MW (2002). Phylogenetic analysis reveals multiple lateral transfer of adenosine-5′-phosphosulfate reductase gene among sulfate-reducing microorganisms. J Bacteriol.

[10] Geets J, Borremans B, Diels L, Springael D, Vangronsveld J, van der Lelie D, Vanbroekhoven K (2006). *DsrB* gene-based DGGE for community and diversity surveys of sulfate-reducing bacteria. J. Microbiol Methods.

[11] Giloteaux L, Goñi-Urriza M, Duran R (2010). Nested PCR and new primers for analysis of sulfate-reducing bacteria in low-cell-biomass environments. Appl Environ Microbiol.

[12] Hall TA (1999). BioEdit: a user-friendly biological sequence alignment editor and analysis program for Windows 95/98/NT. Nucl Acids Symp Ser.

[13] Holmer M, Kristensen E (1996). Seasonality of sulfate reduction and pore water solutes in a marine fish farm sediment: the importance of temperature and sedimentary organic matter. Biogeochemistry.

[14] Japan Fisheries Resource Conservation Association (1995). Environmental Standards for Aquaculture, 1995.

[15] Japan Fisheries Resource Conservation Association (2005). Environmental Standards for Aquaculture, 2005.

[16] Joulian C, Ramsing NB, Ingvorsen K (2001). Congruent phylogenies of most common small-subunit rRNA and dissimilatory sulfite reductase gene sequences retrieved from estuarine sediments. Appl Environ Micorbiol.

[17] Jørgensen BB (1982). Mineralization of organic matter in the sea-bed—The role of sulfate reduction. Nature.

[18] Kawahara N, Shigematsu K, Miura S, Miyadai T, Kondo R (2008). Distribution of sulfate-reducing bacteria in fish farm sediments on the coast of southern Fukui Prefecture, Japan. Plankton Benthos Res.

[19] Kawahara N, Shigematsu K, Miyadai T, Kondo R (2009). Comparison of bacterial communities in fish farm sediments along an organic enrichment gradient. Aquaculture.

[20] Klein M, Friedrich M, Roger AJ, Hugenholtz P, Fishbain S, Abicht H, Blackall LL, Stahl DA, Wagner M (2001). Multiple lateral transfer of dissimilatory sulfite reductase genes between major lineages of sulfate-reducing prokaryotes. J Bacteriol.

[21] Kondo R, Ishida Y, Sugita H (2000). Sulfide. Microbiological Methods for Assessment of Marine Environments.

[22] Kondo R, Nedwell DB, Purdy KJ, Silva SQ (2004). Detection and enumeration of sulphate-reducing bacteria in estuarine sediments by competitive PCR. Geomicrobiol J.

[23] Kondo R, Osawa K, Mochizuki L, Fujioka Y, Butani J (2006). Abundance and diversity of sulphate-reducing bacterioplankton in Lake Suigetsu, a meromictic lake in Fukui, Japan. Plankton Benthos Res.

[24] Kondo R, Butani J (2007). Comparison of the diversity of sulfate-reducing bacterial communities in the water column and the surface sediments of a Japanese meromictic lake. Limnology.

[25] Kondo R, Purdy KJ, Silva SQ, Nedwell DB (2007). Spatial dynamics of sulphate-reducing bacterial compositions in sediment along a salinity gradient in a UK estuary. Microbes Environ.

[26] Kondo R, Shigematsu K, Butani J (2008). Rapid enumeration of sulphate-reducing bacteria from aquatic environments using real-time PCR. Plankton Benthos Res.

[27] Leloup J, Quillet L, Berthe T, Petit F (2006). Diversity of the *dsrAB*(dissimilatory sulfite reductase) gene sequences retrieved from two contrasting mudflats of the Seine estuary, France. FEMS Microbiol Ecol.

[28] Leloup J, Loy A, Knob NJ, Borowski C, Wagner M, Jørgensen BB (2007). Diversity and abundance of sulfate-reducing micro-organisms in the sulfate and methane zones of a marine sediment, Black Sea. Environ Microbiol.

[29] Madrid VM, Aller RC, Aller JY, Chistoserdov AY (2006). Evidence of the activity of dissimilatory sulfate-reducing prokaryotes in nonsulfidogenic tropical mobile muds. FEMS Microbiol Ecol.

[30] Magurran AE (2004). Measuring Biological Diversity.

[31] Mullins TD, Britschgi TB, Krest RL, Giovannoni ST (1995). Genetic comparisons reveal the same unknown bacterial lineages in Atlantic and Pacific bacterioplankton communities. Limnol Oceanogr.

[32] Mußman M, Ishii K, Rabus R, Amann R (2005). Diversity and vertical distribution of cultured and uncultured *Deltaproteobacteria* in an intertidal mud flat of the Wadden Sea. Environ Microbiol.

[33] Nercessian O, Bienvenu N, Moreira D, Prieur D, Jeanthon C (2005). Diversity of functional genes of methanogens, methanotrophs and sulfate reducers in deep-sea hydrothermal environments. Environ Microbiol.

[34] Oakley BB, Carbonero F, van der Gast CJ, Hawkins RJ, Purdy KJ (2010). Evolutionary divergence and biogeography of sympatric niche-differentiated bacterial populations. ISME J.

[35] Pitkänen LK, Tamminen M, Hynninen A, Karkman A, Corander J, Kotilainen A, Virta M (2011). Fish farming affects the abundance and diversity of the mercury resistance gene *merA* in marine sediments. Microbes Environ.

[36] Purdy KJ, Nedwell DB, Embley TM, Takii S (2001). Use of 16S rRNA-targeted oligonucleotide probes to investigate the distribution of sulphate-reducing bacteria in estuarine sediments. FEMS Microbiol Ecol.

[37] Schloss PD, Handelsman J (2005). Introducing DOTUR, a computer program for defining operational taxonomic units and estimating species richness. Appl Environ Microbiol.

[38] Singleton D, Furlong MA, Rathbun SL, Whitman WB (2001). Quantitative comparisons of 16S rRNA gene sequence libraries from environmental samples. Appl Environ Microbiol.

[39] Taketani RG, Yoshiura CA, Dias ACF, Andreote FD, Tsai SM (2010). Diversity and identification of methanogenic archaea and sulphate-reducing bacteria in sediments from a pristine tropical mangrove. Antonie van Leeuwenhoek.

[40] Tamura K, Dudley J, Nei M, Kumar S (2007). MEGA4: Molecular Evolutionary Genetics Analysis (MEGA) software version 4.0. Mol Biol Evol.

[41] Thompson JD, Gibson TJ, Plewniak F, Jeanmougin F, Higgins DG (1997). The CLUSTAL_X windows interface: flexible strategies for multiple sequence alignment aided by quality analysis tools. Nucleic Acids Res.

[42] Thomsen TR, Finster K, Ramsing NB (2001). Biogeochemical and molecular signatures of anaerobic methane oxidation in a marine sediment. Appl Environ Microbiol.

[43] Wagner M, Roger AJ, Flax JL, Brusseau GA, Stahl DA (1998). Phylogeny of dissimilatory sulfite reductases supports an early origin of sulfate respiration. J Bacteriol.

[44] Wenter R, Wanner G, Schüler D, Overmann J (2009). Ultra-structure, tactic behaviour and potential for sulfate reduction of a novel multicellular magnetotactic prokaryote from North Sea sediments. Environ Microbiol.

[45] Widdel F, Balows A, Trüper HG, Dworkin M, Harder W, Schleifer K-H (1992). The genus *Desulfotomaculum*. The Prokaryotes: A Handbook on the Biology of Bacteria: Ecophysiology, Isolation, Identification, Applications.

[46] Yoshida M, Ishii S, Otsuka S, Senoo K (2010). *nirK*-harboring denitrifier are more responsive to denitrification-inducing conditions in rice paddy soil than *nirS*-harboring bacteria. Microbes Environ.

[47] Zhang W, Song L-S, Ki J-S, Lau C-K, Li X-D, Qian P-Y (2008). Microbial diversity in polluted harbor sediments II: Sulfate-reducing bacterial community assessment using terminal restriction fragment length polymorphism and clone library of *dsrAB* gene. Estuar Coast Shelf Sci.

