# Supplementary material for: Comparison of Sulphate-reducing Bacterial Communities in Japanese Fish Farm Sediments with Different Levels of Organic Enrichment
Source: Microbes Environ. 2012 Feb 22;27(2):193–9. doi: 10.1264/jsme2.ME11278 (PMC4036007; doi:10.1264/jsme2.ME11278)
Supplement: Supplementary file 1 [file 27_193_s1.pdf]

Table S1. Assignment of *dsrB* clones from sediment samples of fish farms to distinct operational taxonomic units (OTUs).

OTUs number	Gokasho A2	Shitaba S-7	Yatsushiro A
1	gok001, gok011, gok031, gok033, gok049, gok061, gok104, gok121, gok158, gok175, gok219, gok244, gok253, gok277, gok285, gok304, gok316, gok321, gok343, gok358, gok390, gok425, gok428, gok466	shi032, shi038, shi042, shi046, shi049, shi222, shi239, shi242, shi273, shi278, shi285, shi328, shi354, shi433, shi450	yat092, yat130, yat177, yat187, yat210, yat236, yat288, yat289, yat302
2	gok002, gok035, gok090, gok094, gok095, gok100, gok105, gok136, gok139, gok143, gok187, gok194, gok217, gok274, gok317, gok353, gok363, gok406, gok409, gok410	shi012, shi039, shi047, shi048, shi061, shi074, shi079, shi082, shi095, shi118, shi175, shi177, shi183, shi186, shi190, shi192, shi220, shi225, shi226, shi247, shi259, shi263, shi284, shi295, shi308, shi309, shi317, shi336, shi350, shi356, shi383, shi397, shi428	yat008, yat014, yat016, yat018, yat027, yat030, yat031, yat033, yat041, yat044, yat056, yat061, yat066, yat078, yat082, yat095, yat096, yat131, yat137, yat143, yat148, yat152, yat156, yat160, yat167, yat173, yat184, yat192, yat197, yat198, yat203, yat204, yat208, yat209, yat223, yat230, yat234, yat243, yat254, yat255, yat258, yat263, yat266, yat273, yat280, yat305, yat310, yat320, yat321, yat322, yat324
3	gok003, gok016, gok026, gok060, gok066, gok067, gok073, gok103, gok106, gok107, gok114, gok130, gok181, gok182, gok190, gok196, gok200, gok203, gok204, gok209, gok226, gok271, gok278, gok338, gok346, gok411, gok412, gok416, gok437, gok450, gok460, gok467, gok477	shi003, shi015, shi031, shi035, shi037, shi045, shi062, shi064, shi069, shi077, shi089, shi091, shi092, shi096, shi105, shi107, shi108, shi119, shi142, shi153, shi161, shi196, shi203, shi208, shi212, shi219, shi223, shi227, shi229, shi232, shi245, shi255, shi257, shi269, shi279, shi283, shi298, shi313, shi324, shi335, shi338, shi344, shi351, shi359, shi364, shi372, shi375, shi376, shi399, shi409, shi411, shi413, shi430, shi434, shi444, shi458, shi459	yat019, yat029, yat178, yat200, yat220, yat231, yat244, yat259, yat272, yat290, yat295, yat333
4	gok004, gok005, gok123, gok379, gok413, gok480, gok483	shi102, shi103, shi145, shi166, shi182, shi184, shi246, shi272, shi274, shi301, shi306, shi322, shi329, shi370, shi382, shi401	yat009, yat012, yat039, yat049, yat057, yat059, yat062, yat068, yat100, yat153, yat174, yat188, yat194, yat222, yat232, yat238, yat269, yat277, yat279, yat294, yat314, yat319, yat328, yat330, yat334
5	gok006, gok012, gok015, gok022, gok023, gok042, gok043, gok045, gok069, gok081, gok137, gok149, gok151, gok152, gok155, gok166, gok167, gok169, gok183, gok230, gok243, gok261, gok262, gok265, gok270, gok286, gok322, gok328, gok329, gok331, gok341, gok351, gok404, gok415, gok444		
6	gok007, gok014, gok102, gok109, gok115, gok172, gok178, gok223, gok268, gok288, gok300, gok315, gok380, gok387	shi033, shi303, shi464	yat104
7	gok008, gok038, gok097, gok165, gok227, gok245, gok249, gok256, gok309, gok332, gok342, gok365, gok388, gok430, gok431, gok432, gok439, gok448, gok474, gok481, gok484, gok485	shi460	
8	gok009, gok046, gok080, gok101, gok214, gok242, gok367	shi019, shi067, shi173, shi237, shi291, shi312, shi320, shi355, shi410, shi439	yat020, yat047, yat109, yat110, yat111, yat165, yat181, yat195, yat233, yat312
9	gok010, gok093, gok254, gok375	shi065, shi266, shi315, shi341, shi390, shi403	
10	gok013, gok086, gok292, gok475	shi026, shi387, shi400	
11	gok017, gok020, gok040, gok052, gok116, gok215, gok231, gok246, gok361	shi017, shi043, shi085, shi395	yat142, yat276, yat297

Table S1. Continued.

OTUs number	Gokasho A2	Shitaba S-7	Yatsushiro A
12	gok018		yat249, yat262
13	gok019, gok053, gok457	shi097, shi307, shi316, shi445	yat239, yat257
14	gok021, gok027, gok082, gok117, gok153, gok191, gok213, gok222, gok234, gok276, gok301, gok334, gok360, gok372, gok378, gok403, gok417, gok427, gok434, gok441, gok443, gok449, gok482	shi057, shi115, shi135, shi136, shi250, shi254, shi294, shi357, shi468, shi471	yat002, yat048, yat050, yat052, yat053, yat067, yat091, yat099, yat101, yat129, yat166, yat183, yat211, yat213, yat214, yat218, yat227, yat235, yat247, yat250, yat267, yat271, yat278, yat299, yat303, yat306, yat307, yat308, yat309, yat313, yat315, yat325
15	gok024, gok025, gok030, gok036, gok039, gok050, gok058, gok068, gok071, gok125, gok128, gok131, gok146, gok150, gok156, gok170, gok176, gok184, gok192, gok198, gok201, gok208, gok252, gok260, gok267, gok275, gok299, gok307, gok318, gok325, gok330, gok333, gok337, gok347, gok348, gok354, gok359, gok362, gok377, gok382, gok383, gok385, gok386, gok398, gok400, gok402, gok407, gok414, gok418, gok422, gok429, gok433, gok438, gok440, gok445, gok447, gok462, gok465, gok468, gok471, gok472, gok479, gok486	shi006, shi007, shi023, shi029, shi056, shi060, shi063, shi075, shi078, shi101, shi109, shi111, shi112, shi113, shi114, shi121, shi139, shi141, shi143, shi149, shi154, shi159, shi167, shi168, shi180, shi197, shi198, shi209, shi211, shi215, shi218, shi238, shi243, shi248, shi261, shi280, shi290, shi314, shi323, shi332, shi353, shi361, shi380, shi381, shi414, shi415, shi416, shi418, shi432, shi435, shi438, shi447, shi448, shi451, shi454, shi457, shi461	yat077, yat118, yat140, yat159, yat298, yat301
16	gok028, gok087, gok247	shi214, shi262	
17	gok029, gok193		yat054, yat076, yat139, yat179
18	gok032	shi116, shi331, shi408, shi441	yat025, yat274
19	gok034, gok168, gok364		
20	gok037, gok368		yat284
21	gok041, gok064, gok119, gok122, gok126, gok206, gok225, gok259, gok281, gok290, gok314, gok399	shi059	
22	gok044, gok154, gok264, gok280, gok289, gok469	shi068, shi071, shi081, shi140, shi170, shi172, shi176, shi187, shi188, shi193, shi253, shi326, shi345, shi346, shi396, shi421, shi436	yat074, yat117, yat149
23	gok047, gok323, gok352, gok451	shi174, shi287	
24	gok048, gok065, gok135, gok180, gok224, gok250, gok279, gok319, gok324, gok326, gok339	shi419	yat001
25	gok051, gok054, gok148, gok237, gok269, gok374	shi050, shi122, shi158, shi217, shi470	yat017
26	gok055		
27	gok056		
28	gok057, gok074		
29	gok059, gok110, gok240, gok306, gok356	shi005, shi010, shi025, shi051, shi052, shi070, shi084, shi160, shi200, shi321, shi330, shi334, shi366, shi386, shi412	yat010, yat011, yat024, yat046, yat063, yat072, yat084, yat085, yat086, yat124, yat126, yat134, yat138, yat157, yat162, yat168, yat186, yat189, yat196, yat201, yat237, yat253, yat264, yat282, yat283, yat291, yat292, yat317, yat323
30	gok062	shi134	
31	gok063	shi040	
32	gok070		
33	gok072, gok255, gok394	shi036, shi305, shi392, shi463	yat107, yat185
34	gok075		
35	gok076, gok179		
36	gok077	shi053	

Table S1. Continued.

OTUs number	Gokasho A2	Shitaba S-7	Yatsushiro A
37	gok078	shi008, shi027, shi034, shi131, shi151, shi230, shi235, shi260, shi276, shi282, shi360, shi369, shi389, shi442, shi453, shi466	
38	gok079, gok118, gok420		
39	gok083, gok096		yat088
40	gok084, gok392		
41	gok085		
42	gok088		yat219, yat268
43	gok089		
44	gok091, gok216		yat069
45	gok092, gok303, gok381, gok391	shi002, shi162, shi201, shi234, shi289	yat071
46	gok098	shi191, shi268, shi318, shi449	
47	gok099		
48	gok108		
49	gok111, gok282, gok366	shi030, shi066, shi100, shi125, shi231, shi233, shi348, shi404, shi407, shi417, shi465	yat058, yat169, yat242
50	gok112, gok133, gok160, gok189, gok195, gok197, gok205, gok218, gok357	shi093	yat112
51	gok113		
52	gok120		yat004, yat045, yat064, yat329
53	gok124		
54	gok127, gok157, gok164, gok210, gok297	shi022, shi083, shi347	
55	gok129		
56	gok132, gok257	shi073,, shi087	
57	gok134		
58	gok138, gok159, gok233, gok464	shi016	
59	gok140, gok144, gok370	shi013, shi028, shi299, shi342	yat190, yat286
60	gok141		
61	gok142	shi127	
62	gok145	shi086	yat087, yat215
63	gok147		
64	gok161, gok272, gok310, gok384, gok459	shi011, shi018, shi020, shi130, shi155, shi221, shi224, shi228, shi241, shi244, shi251, shi277, shi292, shi293, shi337, shi343, shi388, shi394	yat065
65	gok162, gok349		yat146
66	gok163		yat224
67	gok171, gok185		
68	gok173		
69	gok174, gok220, gok442	shi094	
70	gok177, gok327	shi327, shi373, shi446	
71	gok186		
72	gok188		
73	gok199, gok251, gok350, gok454	shi443	yat023, yat172
74	gok202		
75	gok207		
76	gok21, gok345		
77	gok212, gok369	shi150, shi311	
78	gok221	shi164,, shi362	yat106, yat164, yat206
79	gok228		
80	gok229, gok453, gok473	shi080, shi106, shi358, shi363, shi368	yat136
81	gok232		

Table S1. Continued.

OTUs number	Gokasho A2	Shitaba S-7	Yatsushiro A
82	gok235	shi044, shi146, shi171, shi349, shi424	yat150
83	gok236		
84	gok238		
85	gok239		
86	gok241	shi216, shi236	yat125
87	gok248		
88	gok258, gok455, gok456	shi352,, shi367, shi378	
89	gok263		
90	gok266		
91	gok273, gok302	shi169, shi297, shi398	yat116
92	gok283		
93	gok284, gok340		
94	gok287		
95	gok291, gok419		
96	gok293, gok312, gok336	shi185, shi296	yat300
97	gok294, gok298		
98	gok295, gok296		
99	gok305		
100	gok308	shi339	
101	gok311		yat043, yat147, yat163, yat285
102	gok313		
103	gok320	shi098, shi099, shi393	yat006, yat022, yat051, yat073, yat122, yat144, yat191, yat217, yat245, yat251, yat261, yat287, yat326, yat327, yat331
104	gok335		
105	gok344		
106	gok355		
107	gok371	shi088, shi300	yat103
108	gok373	shi024, shi365	yat032, yat105
109	gok376		yat226
110	gok389	shi205	
111	gok393		
112	gok395		
113	gok396		yat318
114	gok397		yat296
115	gok401, gok446, gok452, gok458	shi004, shi014, shi144, shi371	
116	gok405		
117	gok408	shi021, shi055, shi058, shi165, shi206, shi402, shi420	yat123, yat202, yat260
118	gok421		
119	gok423	shi054, shi110, shi207	yat005
120	gok424	shi041, shi076, shi319, shi469	
121	gok426	shi001, shi310	
122	gok435		
123	gok436	shi210	yat114
124	gok461		
125	gok463		
126	gok470		
127	gok476		
128	gok478		
129		shi009, shi202	yat013
130		shi072	yat132
131		shi090, shi157	
132		shi104, shi256, shi271	yat265
133		shi117	yat040, yat113, yat119, yat180

Table S1. Continued.

OTUs number	Gokasho A2	Shitaba S-7	Yatsushiro A
134		shi120	
135		shi123	
136		shi124	
137		shi126	yat120
138		shi128, shi179, shi429, shi437	yat083
139		shi129	
140		shi132, shi406	
141		shi133, shi137, shi147, shi204, shi252, shi391	yat182
142		shi138, shi302	
143		shi148, shi178, shi456	yat108
144		shi152, shi427	yat155, yat171
145		shi156	
146		shi163	
147		shi181, shi275	
148		shi189	
149		shi194, shi270, shi377, shi384	
150		shi195, shi467	yat293
151		shi199, shi240, shi288	yat026
152		shi213	
153		shi249, shi258, shi264	
154		shi265	
155		shi267	yat176, yat275
156		shi281	
157		shi286	
158		shi304	
159		shi325	
160		shi333	
161		shi340, shi452	
162		shi374	
163		shi379	
164		shi385	yat081
165		shi405	
166		shi422	
167		shi423, shi426	yat007
168		shi425	
169		shi431	
170		shi440	
171		shi455	
172		shi462	
173			yat003, yat015
174			yat021, yat090, yat229, yat097
175			yat028
176			yat034
177			yat035, yat154, yat252, yat175, yat270
178			yat036
179			yat037
180			yat038
181			yat042
182			yat055
183			yat060
184			yat070, yat221
185			yat075
186			yat079, yat240
187			yat080

Table S1. Continued.

OTUs number	Gokasho A2	Shitaba S-7	Yatsushiro A
188			yat089
189			yat093, yat094
190			yat098, yat216
191			yat102
192			yat115, yat281
193			yat121
194			yat127
195			yat128
196			yat133
197			yat135
198			yat141
199			yat145
200			yat151
201			yat158
202			yat161
203			yat170, yat311
204			yat193
205			yat199
206			yat205
207			yat207
208			yat212
209			yat225
210			yat228
211			yat241
212			yat246
213			yat248
214			yat256
215			yat304
216			yat316
217			yat332
